# Effects of Maturity and Processing on the Volatile Components, Phytochemical Profiles and Antioxidant Activity of Lotus (*Nelumbo nucifera*) Leaf

**DOI:** 10.3390/foods12010198

**Published:** 2023-01-02

**Authors:** Zhili Ma, Yu Ma, Yin Liu, Bei Zhou, Yalin Zhao, Ping Wu, Dexin Zhang, Deyuan Li

**Affiliations:** 1 School of Laboratory Medicine, Hubei University of Chinese Medicine, Wuhan 430065, China; 2 Wuhan Huanghelou Essence and Flavor Co., Ltd., Wuhan 430040, China; 3 College of Food Science and Technology, Huazhong Agricultural University, Wuhan 430070, China

**Keywords:** lotus leaf, tea, processing, volatiles, GC-IMS, phenols, antioxidant activity

## Abstract

In this study, fresh lotus leaves at two maturity stages were processed to tea products by different methods (white-tea process, green-tea process and black-tea process). The volatile compounds, phytochemical profiles and antioxidant activities of lotus-leaf tea were investigated. A total of 81 volatile components were identified with HS-GC-IMS. The mature lotus-leaf tea showed more volatile compounds than the tender lotus-leaf tea. The lotus leaf treated with the white-tea process had more aroma components than other processing methods. In addition, six types of phenolic compounds, including luteolin, catechin, quercetin, orientin, hyperoside and rutin were identified in the lotus-leaf tea. The mature leaves treated with the green-tea process had the highest levels of TPC (49.97 mg gallic acid/g tea) and TFC (73.43 mg rutin/g tea). The aqueous extract of lotus-leaf tea showed positive scavenging capacities of DPPH and ABTS radicals, and ferric ion reducing power, whereas tender lotus leaf treated with the green-tea process exhibited the strongest antioxidant activity. What is more, the antioxidant activities had a significant positive correlation with the levels of TPC and TFC in lotus-leaf tea. Our results provide a theoretical basis for the manufacture of lotus-leaf-tea products with desirable flavor and health benefits.

## 1. Introduction

*Nelumbo nucifera Gaertn*., also called lotus, is cultivated in many parts of China. The annual yield of lotus leaves is 70–100 M tons but only 1% is utilized in the food industry in China [1]. Lotus leaf (also called “HeYe”) was listed as a food and medicine resource by the Ministry of Health of China in 1991 [2]. Many pharmacological and physiological activities have been reported in lotus leaves, including anti-obesity [3,4], hemostatic action [5], enhancement of sleep quality [6], prevention of atherosclerosis [7], prevention of Alzheimer’s Disease [8], anti-cancer [9], anti-inflammatory [10], and modulation of immune response and intestinal microbiota composition [11]. Additionally, the antioxidant activity has also been confirmed [12,13,14]. Lotus leaf powder is also reported to have been used for decreasing oxidation and maintaining color stability in cooked pork during the cold storage [15].

It has been reported that the active ingredients in lotus leaves are alkaloids, flavonoids, phenolic acids, terpenoids and polysaccharides [16]. However, the bioactivities of lotus leaves are mainly conferred by phenolic compounds, such as phenolic acids and flavonoids [17] and alkaloids [18]. The identified phenolic compounds are reported to be catechin, myricetin, isoquercetin, hyperin and kaempferol [16].

Aroma is an important factor in the quality evaluation of tea products. Hundreds of volatile compounds in teas have been reported. The main aroma components are aldehydes, ketones, alcohols and heterocyclic compounds [19]. In Han et al.’s study, with pan-fire treatment and steam treatments, there was a difference in the monoterpenol-related floral scent in green tea [20]. Wei et al. found that the flavor of summer green tea was improved by the yellowing process [21]. It has also been reported that different drying temperatures can cause different aroma compounds in green teas [22]. For black tea, the levels of volatile components increased in the fermentation stage but decreased in the drying stage [23]. In summary, different tea manufacturing methods affect the odors of the tea, and the drying temperature and fermentation treatment are the major factors [24]. In addition, the fresh leaves from different seasons and different tea trees have an effect on the aroma of teas [25,26]. Similar results were observed in some other food materials. In *Lentinula edodes*, the volatile components were influenced by the drying process and the aroma profiles of banana were affected by different maturity and cultivars [27,28]. Additionally, different types of foods have their own specific aroma profiles, such as ester compounds for banana fruits and sulfur compounds for *Lentinula edodes*. For volatiles analysis, GC-E-Nose, GC-IMS and GC-MS were the dominant analysis methods [29,30]. Although there is limited literature on lotus-leaf-tea volatiles, aroma indeed influences tea quality and consumers’ preference. Furthermore, lotus leaf had a special fragrance, and revealing the flavor features of lotus leaf may contribute to a better understanding of the quality of the lotus-leaf tea.

There have been few studies on the effects of the maturity of leaves and the processing method on flavor, bioactive profiles and antioxidant capacities of lotus (*Nelumbo nucifera*) leaf. The objective of this study was to prepare lotus-leaf tea by the white-tea process, the green-tea process and the black-tea process using fresh leaves at tender and mature growth stages. The flavor and phytochemical profiles of different types of lotus-leaf tea were investigated. The antioxidant functions including scavenging capacities of DPPH and ABTS radicals, and ferric ion reducing power were also determined.

## 2. Materials and Methods

### 2.1. Materials and Chemicals

Fresh lotus leaves were purchased from Ganzhou, Jiangxi province, China. Space lotus-36 was cultivated in a sheltered and sunny paddy field with fertile soil and sufficient water. The thickness of the soil layer was approximately 30 cm. The lotus root seed was planted in March. The tender lotus leaves were grown for 2–3 days, whereas the mature lotus leaves grown for 5–7 days. 1,1-diphenyl-2-picryl-hydrazyl (DPPH, CAS 1898-66-4) radical, 2,2′-azino-bis-3-ethylbenzthiazoline-6-sulphonic acid (ABTS^+^, CAS 30931-67-0) radical, gallic acid monohydrate (CAS 149-91-7), rutin (CAS 153-18-4) and Folin–Ciocalteu reagent (CAS 12111-13-6) were obtained from Shanghai Yuanye Bio-Technology Co., Ltd. (Shanghai, China). Other chemicals of analytically purity were purchased from Sinopharm Chemical Reagent Co., Ltd. (Beijing, China).

### 2.2. Preparation of Lotus-Leaf Tea and Aqueous Extract

**Figure 1:**
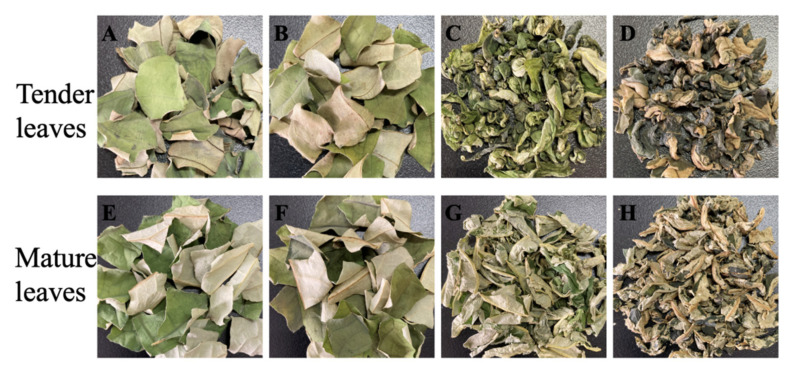
The appearance of lotus-leaf teas with different processing methods. (**A**) TNT, (**B**) THT, (**C**) TGT, (**D**) TBT, (**E**) MNT, (**F**) MHT, (**G**) MGT, (**H**) MBT.

The fresh lotus leaves at different growth stage were processed by four processing methods. The diameters of tender and mature lotus leaves were 15–20 and 45–50 cm, respectively. Fresh lotus leaves were withered at 28 °C overnight. After that, the leaves were cut into pieces of 2 × 2 cm. Then, the pieces were divided equally into four portions followed by four processing methods (Figure 1). Group A (tender naturally-dried lotus-leaf tea, TNT) and Group E (mature naturally-dried lotus-leaf tea, MNT) were naturally dried at 30 °C, RH 70%. Group B (tender hot-air-dried lotus-leaf tea, THT) and Group F (mature hot-air-dried lotus-leaf tea, MHT) were hot air dried at 80 °C. Groups A, B, E and F were made by the white-tea process. Group C (tender green lotus-leaf tea, TGT) and Group G (mature green lotus-leaf tea, MGT) were microwave-treated for 2 min, kneaded into cords, and dried at 80 °C, which was the green-tea process. Group D (tender black lotus-leaf tea, TBT) and Group H (mature black lotus-leaf tea, MBT) were kneaded into cords, fermented at 27 °C, RH 90% for 5 h, and then dried at 80 °C, which was the black-tea process. The water content of all the lotus-leaf teas was approximately 4% (*w*/*w*). 

The lotus-leaf teas were ground into powder, and 1.0 g of the samples was put into a 200 mL conical flask, and then soaked in hot water at 1:20 ratio (*w*/*v*) in a 80 °C water bath for 10 min. After centrifugation, the supernatant was freeze-dried to obtain the aqueous extract.

### 2.3. Determination of Volatile Compounds

The FlavourSpec^®^ (G.A.S., Dortmund, Germany) system was used for gas chromatography–ion mobility spectrometry (GC-IMS) analysis. Two grams of lotus-leaf tea sample was introduced into a 20 mL headspace vial and incubated for 10 min at 60 °C. Then, the samples were injected into an MXT-5 capillary column (15 m × 0.53 mm, 1.0 μm) with a column temperature of 60 °C. The flow rate was as follows: initial flow rate 2 mL/min, hold for 2 min, and ramp up to 10 mL/min at the time of 10 min, then an increase to 100 mL/min at the time of 25 min, hold for 5 min. The condition of the IMS ionization chamber was a 3H ionization source with a nitrogen flow rate of 150 mL/min. The drift tube length was 98 mm with a constant voltage of 5 kV at 45 °C.

All analyses were performed in duplicate. The analysis software Vocal, which is equipped with a GC-IMS instrument, was used for data collection and analysis. Volatile compounds were identified using the RI and drift time to standards in the GC-IMS library. The built-in Reporter and Gallery Plot plug-ins of the software were used to draw two-dimensional maps and fingerprints of volatile components of the samples.

### 2.4. Determination of Total Phenolic and Flavonoid Content

Total phenolic content (TPC) was determined using Folin–Ciocalteu reagent with a reported method [31]. In brief, each 50 μg/mL of the aqueous extract was prepared using ddH_2_O. First, 20 μL of gallic acid standard or sample was reacted with 100 μL of 10-fold diluted Folin–Ciocalteu reagent for 1 min. After 30 min, 80 μL of 75 mg/mL Na_2_CO_3_ was added. The 765 nm absorbance was obtained by a Multiskan Ascent spectrophotometer (Thermo Labsystems, Helsinki, Finland). Values of TPC are shown as μg gallic acid/g lotus-leaf tea.

Total flavonoid content (TFC) was measured according to a reported method [32]. Briefly, each 1.0 mg/mL of the aqueous extract was prepared with ddH_2_O. First, 1 mL of rutin standard or sample was mixed with exactly 1 mL of NaNO_2_ solution (5%). After that, 1 mL of AlCl_3_·6H_2_O (10%) and 5 mL of NaOH (4%) were added to the mixture for a 6 min incubation. Then, 17 mL of ddH_2_O was added and 5 min later the absorbance of 510 nm was measured. TFC is shown as μg rutin/g lotus-leaf tea.

### 2.5. Determination of Antioxidant Activity

#### 2.5.1. DPPH Radical Scavenging Activity

The scavenging capacities of DPPH free radicals of aqueous extract samples were assessed with our reported method [33]. Briefly, different concentrations of aqueous extract were reacted with 0.1 mM DPPH for 10 min at 25 °C. After that, the absorbance was assayed at 519 nm. The percentage inhibition was calculated as % Inhibition = (A_0_ − A_1_)/(A_0_ − A_2_) × 100, the absorbance of 0.1 mM DPPH alone in methanol was A_0_, the absorbance of 0.1 mM DPPH with sample in methanol was A_1_, and the absorbance of methanol solvent alone was A_2_.

#### 2.5.2. ABTS^+^ Radical Scavenging Activity

The scavenging capacities ABTS^+^ free radicals of aqueous extract samples were determined using our reported method [33]. Each of 20 μL of samples at varied concentrations was reacted with 180 μL of ABTS^+^ solution for 10 min at 25 °C. After that, the absorbance at 734 nm was assayed. The percentage inhibition was obtained in the same way as in Section 2.5.1.

#### 2.5.3. Ferric Reducing Power

The ferric ion reducing antioxidant power (FRAP) was determined using our previous method [33]. To prepare the FRAP working solution, 20 mM of FeCl_3_ solution, the TPTZ solution (10 mM), and acetate buffer (pH 3.6, 300 mM) were mixed with a volume ratio 10:1:1. Each of 5 μL sample solution was reacted with 180 μL FRAP solution for 6 min at 25 °C, and FeSO_4_ solutions at different concentrations were used as standards. The absorbance of various incubation solution was measured at 593 nm. The FRAP values of different types of lotus-leaf tea were expressed as equivalent concentrations of FeSO_4_ (mM Fe^2+^/mL).

### 2.6. Determination of Phenolic Compounds by UHPLC-MS/MS

The Waters ACQUITY UHPLC H-Class system (USA) with a Waters ACQUITY UHPLC BEH C18 column (2.1 × 100 mm, 1.7 μm) (Milford, MA, USA) was employed to fractionate the polyphenols in lotus-leaf tea. The mobile phases A was 0.1% formic acid solution and mobile phases B was acetonitrile with a flow rate of 0.35 mL/min. The gradient program was performed as: 0–1.0 min, 90% A and 10% B; 8 min, 85% B; 13 min, 85% B; and 13.0–15.0 min, 90% A and 10% B. The column temperature was 30 °C with an injection volume of 1 μL.

The UHPLC system was coupled to a Vion^®^ ion mobility spectrometer quadrupole-time-of-flight mass spectrometer (IMS Q-TOF MS, Waters, MA, USA). Mass spectrometry conditions: ESI^−^ ion source, capillary voltage was 2.5 kV, source temperature was 135 °C, desolvation temperature was 350 °C, cone gas flow was 50.0 L/h, desolvation gas flow was 600.0 L/h, and collision RF voltage was 20–30 eV. Spectra were recorded over the mass/charge (*m/z*) range of 50–1000. The UNIFI software (Waters, MA, USA) was used to process the MS data to identify polyphenols.

### 2.7. Statistical Analysis

The results are shown as mean ± standard deviation (SD). Differences among the mean values were presented by Duncan’s Multiple Range Test (*p* < 0.05).

## 3. Results and Discussion

### 3.1. Volatile Compounds of Lotus-Leaf Tea by GC-IMS Analysis

**Figure 2:**
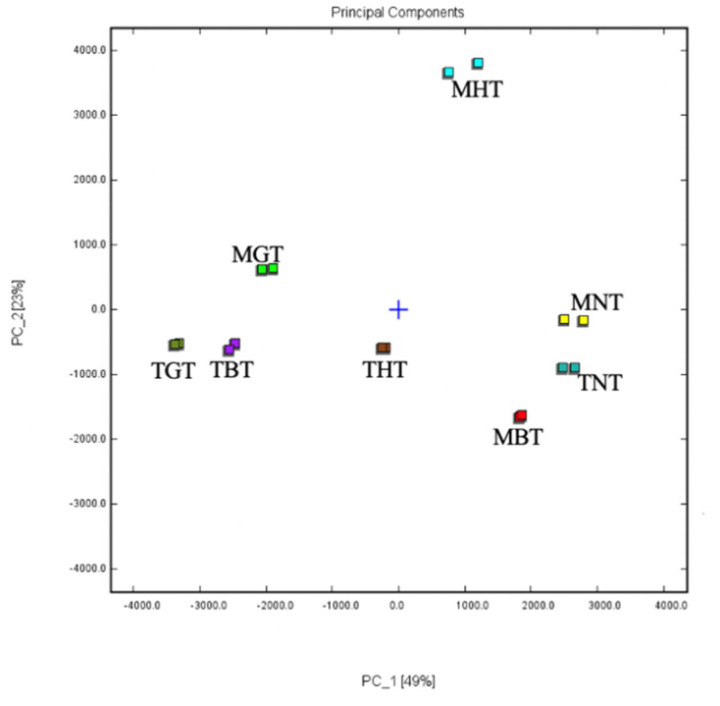
PCA of volatile compounds of eight types of lotus-leaf tea detected via HS-GC-IMS. TNT, tender naturally-dried lotus-leaf tea; THT, tender hot air-dried lotus-leaf tea; TGT, tender green lotus-leaf tea; TBT, tender black lotus-leaf tea; MNT, mature naturally-dried lotus-leaf tea; MHT, mature hot air-dried lotus-leaf tea; MGT, mature green lotus-leaf tea; MBT, mature black lotus-leaf tea.

It was reported that aroma precursors derived from protein, carotenoids, glycosides and lipid increased when the water content of tea leaf decreased [34]. Therefore, abundant aroma components in lotus-leaf tea were produced when treated with withering and drying. The volatile components in different types of lotus-leaf tea were measured via HS-GC-IMS. As shown in Figure 2, the PCA was carried out based on the aroma component data to reveal the similarity among the different lotus-leaf teas. The principal compounds PC1 and PC2 represented 49% and 25% of the total variance, respectively, with the cumulative contribution rate of the first two PCs accounting for 74%. The results indicated that PCA was sufficient for the total variance in the dataset. A clear separation among samples with different processing method was observed, which indicated that flavor analysis can be used for differentiating the types of lotus-leaf teas. Interestingly, the tender naturally dried lotus-leaf tea was close to the mature naturally dried lotus-leaf tea, which indicated a minor difference of naturally dried lotus-leaf teas under different maturities.

**Figure 3:**
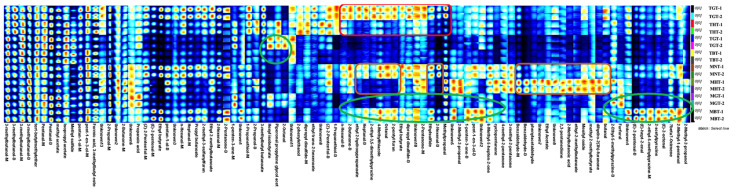
Fingerprints of eight types of lotus-leaf-tea samples. Each column represents a compound. Two parallels in each group. (“M”, monomer and “D”, dimer) detected via HS-GC-IMS.

A total of 81 compounds were identified, including 20 aldehydes, 17 alcohols, 15 esters, 12 ketones, 6 heterocyclic compounds, 4 sulfur ethers, 3 thiols, 2 acids, 1 ether and 1 alkene. Aldehydes, alcohols and esters were the dominant volatiles of the total volatiles, accounting for approximately 64.2%. Notably, more components were observed in the mature groups than that of tender groups, which were mainly esters, aldehydes and ketones. Zuo et al. found a positive correlation between leaf cuticular wax loading and aroma formation [35]. Consistent with this conclusion, the mature groups had more leaf cuticular wax loading than the tender groups and thus, more components than the tender groups. From the fingerprint spectrum in Figure 3, we could also conclude that the naturally dried lotus leaf possessed more volatile organic compounds than the hot-air dried lotus-leaf tea, which mainly were aldehydes and ketones, sulfur-containing compounds and heterocyclic compounds in tender lotus-leaf tea. The aldehydes were methylpropanal, 2-hexenal, octanal and heptanal. The ketones were 2-pentanone and 2-butanone. The sulfur-containing compounds were ethylsulfide, dipropyl disulfide and 1-propanethiol. The heterocyclic compounds included 4-methylthiazole, 2-pentyl furan and 2-ethyl-3,5-dimethylpyrazine. Compared with the “black lotus tea”, the “green lotus tea” from tender leaves had more 2-octanol, piperonal propylene glycol aceta and hexyl isobutyrate. Additionally, 2-octanol was only detected in tender groups.

By contrast, in the mature groups, the naturally dried lotus-leaf tea possessed three types of aldehydes, which were heptanal, octanal and 2-hexenal and three types of heterocyclic compounds, which were 4-methylthiazole, 2-pentyl furan and 2-ethyl-3,5-dimethylpyrazine. Compared with naturally dried lotus-leaf tea, several esters, aldehydes, ketones, and one type of heterocyclic compound and acid were identified in hot-air dried lotus leaf samples. These were: dihydro-2(3h)-furanone, methyl 2-methylbutyrate, ethyl-2-methylbutanoate, ethyl butyrate, butanal, benzaldehyde, phenylacetaldehyde, mesityl oxide, 2,3-pentanedione, 2-ethyl-5-methylpyrazine and 2-methylbutanoic acid. However, Shi et al. reported that the content of benzaldehyde among green, black and white tea from purple-colored leaves was black tea > white tea > green tea [36]. The result was different from ours, which might have been due to the different content of the precursors of benzaldehyde. In addition, there were more volatile components in the “black lotus tea” group than that of “green lotus tea” group, which were six aldehydes, three alcohols, one ketone, one ester, one alkene and five heterocyclic compounds. The six aldehydes were (E)-2-octenal, (E)-hept-2-enal, (E)-2-pentenal, 2-hexenal, octanal and heptanal. Three alcohols were 2-methyl-2-propanol, 2-methyl-1-pentanol and n-hexanol. The ketone was 1-penten-3-one. The ester was ethyl 2-hydroxypropanoate. The alkene was beta-Ocimene, and the 5 heterocyclic compounds were 2-acetylpyrazine, 2-ethyl-5-methylpyrazine, 2-pentyl furan, 4-methylthiazole and 2-ethyl-3,5-dimethylpyrazine.

Aroma molecules in teas differ largely depending on the manufacturing process [29]. As illustrated in Figure 3, for both tender and mature lotus-leaf tea, the black-tea-process and green-tea-process groups had fewer aroma components than white-tea-process group, which might have been caused by the fermentation or microwave treatment. The post-fermentation technologies were reported to influence the volatile profiles of dark teas in Ma et al.’s study [37]. The fixation method also had remarkable effects on the sensory quality of green tea. In a related study, in green teas, microwave fixation was reported to improve the final color of the tea, but with a reduced aroma [38]. Interestingly, according to the results, the effect of the processing method on the volatile compound for tender leaves and mature leaves did not show a similar trend, which might have been due to the different characteristic compounds in two types of lotus-leaf teas with different maturities. It has reported that aroma compounds in teas are generated from carotenoid, lipid, and glycosides precursors and the Maillard reaction [19]. Thus, we concluded that the aroma type of a lotus-leaf tea could be attributed to the different compositions caused by different maturities and processing methods. It has also been reported that the processing technology influenced the aroma type of green tea, which was consistent with our study [20].

### 3.2. Determination of TPC, TFC and Antioxidant Capacity

#### 3.2.1. Analysis of TPC and TFC

TPC and TFC were determined for lotus-leaf teas with different maturities in different processing methods. As shown in Table 1, TPC and TFC of different groups differed significantly at *p* < 0.05. Comparing tender leaves with mature leaves, TPC and TFC of the white-tea process of tender leaves were higher than that of mature leaves. In Su et al.’s study, TPC and TFC from fresh lotus leaf extracts had the similar variations across maturities [12]. TPC of MGT and MBT were 49.97 and 27.51 mg gallic acid/g lotus tea, which were higher than those of TGT (47.45 mg gallic acid/g lotus tea) and TBT (18.65 mg gallic acid/g lotus tea). Similarly, TFC of MGT was 73.43 mg rutin/g lotus tea, which was higher than that of TGT (65.78 mg rutin/g lotus tea).

Relatively, TPC and TFC of green-tea process groups were higher than most of other processing method for either tender leaves or mature leaves, while the black-tea process had the lowest TPC and TFC among the different processing methods. This result was consistent with the teas processed by coffee leaves [31]. The green-tea process from mature leaves had the highest level of TPC (49.97 mg gallic acid/g lotus tea) and TFC (73.43 mg rutin/g lotus tea).

Comparing the naturally dried method with the hot-air-dried method, the tender leaves and mature leaves showed an opposite trend. For the tender leaves, the contents of total polyphenol and total flavonoids of hot-air-dried processing were higher than those of the naturally dried processing method. However, for the mature leaves, the contents of total polyphenol and total flavonoids of hot-air-dried processing were lower than that of the naturally dried processing method.

#### 3.2.2. Analysis of DPPH, ABTS^+^ Radical Scavenging Activities and Ferric Reducing Power

**Figure 4:**
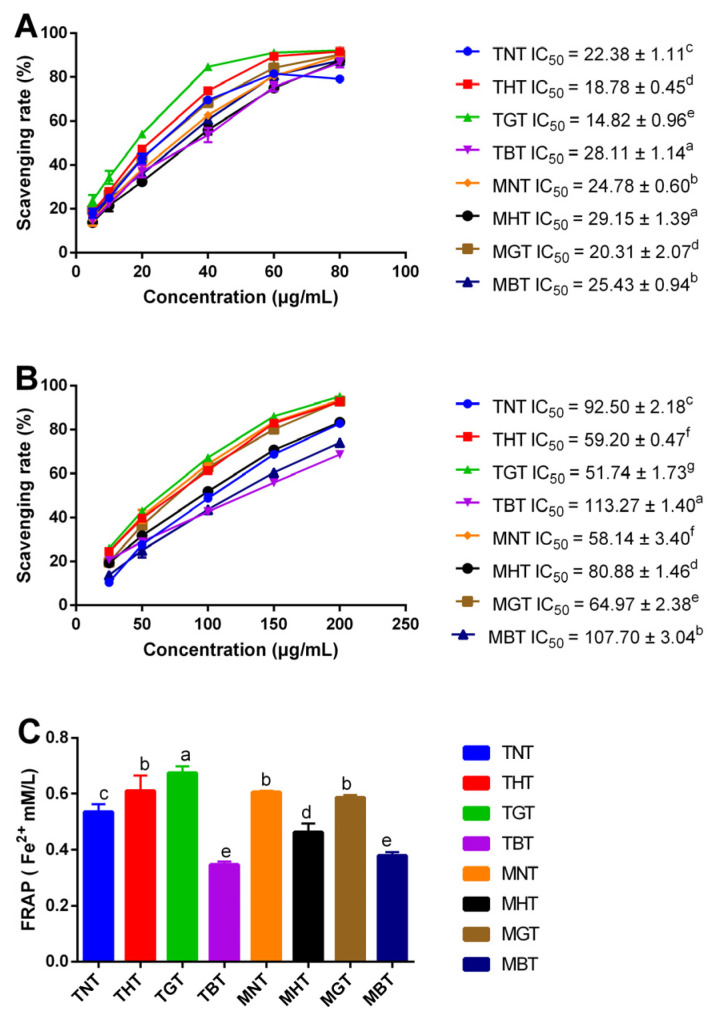
The antioxidant of lotus-leaf tea. (**A**) The capacities of DPPH radicals of eight lotus-tea extractions. (**B**) The capacities of ABTS^+^ radicals of eight lotus-tea extractions. (**C**) FRAP abilities of eight lotus tea extractions. Different letters (a–g) indicate significant differences (*p* < 0.05).

DPPH, ABTS^+^ radical scavenging abilities and FRAP of eight lotus-leaf tea extractions were determined in the present study. As shown in Figure 4A, DPPH radical scavenging rates of the eight types of lotus-leaf tea extractions increased to the peak rate at the concentration of 60 μg/mL, and then the scavenging rates remained steady. IC_50_ values of TNT, THT, TGT, TBT, MNT, MHT, MGT and MBT were 22.38, 18.78, 14.82, 28.11, 24.78, 29.15, 20.31 and 25.43 μg/mL, respectively. For the tender leaves, the scavenging activity increased in the order of: TGT > THT > TNT > TBT, and for the mature leaves, the scavenging activity increased in the order of: MGT > MNT > MBT > MHT. For both tender and mature leaves, IC_50_ of green-tea process was lower than that of black-tea process, which meant the DPPH radical scavenging ability of green-tea-processed lotus-leaf tea was better than that of black-tea-processed lotus-leaf tea. DPPH radical scavenging ability of TGT was better than MGT. Moreover, at a concentration of 60 μg/mL, DPPH radical scavenging rates of TNT, THT, TGT, TBT, MNT, MHT, MGT and MBT reached 81.53%, 89.55%, 91.23%, 75.64, 80.45%, 75.08%, 84.17% and 80.82%, respectively. Our results thus indicated that eight lotus tea extractions had positive scavenging capacities on the DPPH radical and green-tea-processed lotus-leaf tea had a better scavenging capacity than that of black-tea-processed lotus-leaf tea.

ABTS^+^ radical scavenging abilities of eight lotus-leaf-tea extractions are shown in Figure 4B. ABTS^+^ radical scavenging rates of eight lotus-leaf tea extractions increased steadily from the concentration of 25 μg/mL to 200 μg/mL. IC_50_ values of TNT, THT, TGT, TBT, MNT, MHT, MGT and MBT were 92.50, 59.20, 51.74, 113.27, 58.14, 80.88, 64.97 and 107.70 μg/mL, respectively. For the tender leaves, the ABTS^+^ radical scavenging activity increased in the order of: TGT > THT > TNT > TBT, which was consistent with DPPH radical scavenging rate, and for the mature leaves, the ABTS^+^ radical scavenging activity increased in the order of: MNT > MGT > MHT > MBT. As shown in Figure 4B, for both tender and mature leaves, IC_50_ of green-tea process was lower than that of black-tea process, which meant ABTS^+^ radical scavenging ability of green-tea-processed lotus-leaf tea was better than that of black-tea-processed lotus-leaf tea. ABTS^+^ radical scavenging ability of TGT was better than MGT. At the concentration of 200 μg/mL, the ABTS^+^ radical scavenging rates of TNT, THT, TGT, TBT, MNT, MHT, MGT and MBT were 82.76%, 92.75%, 95.09%, 68.71%, 93.46%, 83.38%, 93.06% and 74.16%, respectively. Our results indicated that the various lotus-leaf tea extractions had strong anti-radical activity on the ABTS^+^ radical.

The FRAP of the eight lotus-leaf tea extractions are shown in Figure 4C. FRAP values of TNT, THT, TGT, TBT, MNT, MHT, MGT and MBT were 0.54, 0.61, 0.67, 0.35, 0.61, 0.46, 0.59 and 0.38 mMFe^2+^/L, respectively. Our results indicated that FRAP of the four tender lotus-leaf-tea extractions increased in the order of: TGT > THT > TNT > TBT (*p* < 0.05), which was consistent with DPPH and ABTS^+^ radical scavenging abilities. FRAP of the four mature lotus-leaf tea extractions increased in the order of: MNT > MGT > MHT > MBT (*p* < 0.05), which was consistent with ABTS^+^ radical scavenging ability. Our results suggested that the lotus-leaf tea had potential FRAP activities.

#### 3.2.3. Cluster and Correlation Analysis between TPC, TFC and the Antioxidant Capacity

**Figure 5:**
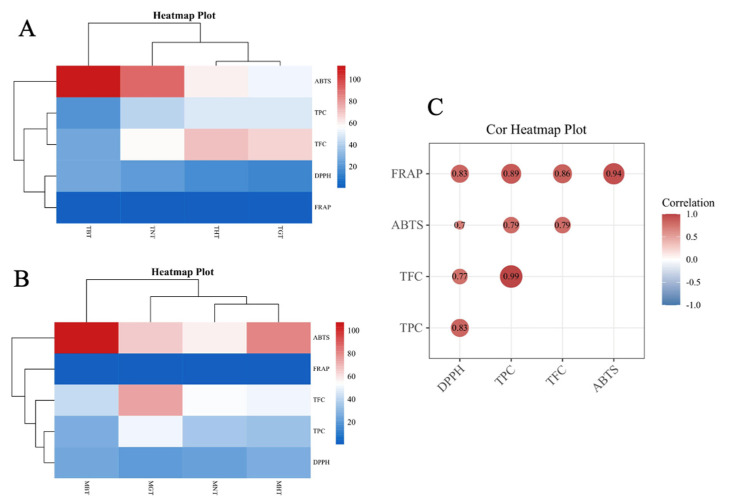
Dendrogram, heatmap plot and correlation heatmap plot of antioxidant activities in different maturities of lotus-leaf-tea samples were based on statistical analysis. (**A**) Dendrogram and heatmap plots of antioxidant activities of tender lotus-leaf-tea samples. (**B**) Dendrogram and heatmap plots of antioxidant activities of mature lotus-leaf-tea samples. (**C**) Correlation heatmap plots of antioxidant index in the lotus-leaf-tea samples.

As shown in Figure 5A, heatmap plots and cluster analyses were used to explore the relationships between processing methods and antioxidant properties. Cluster analysis can classify different processing methods into different branches according to TPC, TFC and the antioxidant capacity assays. The cluster analysis was based on the processing method presented black-tea process samples as individual branches in both tender and mature groups. In tender groups, the TBT was an individual group, while the THT and TGT groups were closer. As shown in Figure 5B, it showed that the MBT was an individual group, the MGT group was an individual group, and the naturally dried and hot-air-dried process were close groups in mature leaves.

As shown in Figure 5C, positive correlations were found among TPC, TFC, DPPH and ABTS^+^ radical scavenging activities and ferric reducing power based on the Pearson test. The highest correlation value was estimated in TPC and TFC (r^2^ = 0.99); similar results were also reported in lotus leaves [12]. DPPH, ABTS^+^ radical scavenging capacities and FRAP were well correlated with TFC (r^2^ = 0.83, 0.79 and 0.89, respectively), but their correlation coefficients were slightly lower than that of TPC (r^2^ = 0.77, 0.79 and 0.86, respectively). In Su et al.’s study [12], the correlation coefficients of TFC with ABTS^+^ (r^2^ = 0.952) radical scavenging activities and FRAP (r^2^ = 0.813) were slightly lower than those of TPC with ABTS^+^ (r^2^ = 0.979) and FRAP (r^2^ = 0.883). Their result was a little different from ours, which might have been caused by the types of different polyphenols or flavonoids. It has been reported that DPPH assay had better correlation with TPC, whereas ABTS^+^ and reducing power had a relatively weak correlation [32]. From their study, the correlation results showed that the main components of the extracts were an important reason for the different correlation. The FRAP showed a relatively high r^2^-value with DPPH (r^2^ = 0.83) and ABTS^+^ (r^2^ = 0.94) radical scavenging activities. The r^2^-values of DPPH and ABTS^+^ radical scavenging activities was 0.70, indicating a relatively weak correlation. According to the statistical analysis results, we found the significant correlation between the levels of phenolic compounds in lotus-leaf-tea extractions and their antioxidant capacities.

### 3.3. Identification of Phenolic Compounds in Lotus-Leaf Tea

In summary, we identified six kinds of phenolic compounds in the lotus-leaf tea samples: luteolin, catechin, quercetin, orientin, hyperoside and rutin. As shown in Table 2, compound 1 (RT = 4.33 min) showed a molecular weight of 286.04. The main fragments at *m*/*z* of 151.00 and 133.06 were formed by retro Diels–Alder reaction from compound 1, which was the most common reaction in MS detection. The fragments were consistent with the results reported by Wu et al. [39]. Compound 2 (RT = 3.01 min) with the molecular weight of 290.07 and the fragment at *m/z* of 245.89, which was the result of neutral loss of CO_2_, was identified as catechin [40]. Catechin in lotus leaves was also reported in some research [41,42]. Compound 3 (RT = 4.08 min) with a molecular weight of 302.03, and the main fragments at *m/z* of 273.06, 257.04 and 179.03 was also reported in a previous study [43]. Therefore, compound 3 was identified as quercetin. Compound 5 (RT = 4.07 min) showed a molecular weight of 464.09, and the main fragment at *m*/*z* of 301.05 was the loss of the monomeric unit of glucose or galactose. Similar results were also reported by Huang et al. [44]. Compound 6 (RT = 3.95 min) showed a molecular weight of 609.14, and the main fragments at *m*/*z* of 301.05 was the loss of a monomeric unit of rhamnose and rutinose [45]. Compound 6 was identified as rutin; rutin was also reported by HPLC or HPLC/MS in the research on the lotus leaf [42,46]. Interestingly, compound 4 with a molecular weight of 448.09 was identified as orientin which has rarely been reported in current research. The fragment with *m/z* of 327.08 was formed by the loss of a neutral fragment of molecular weight of 120 [47].

**Figure 6:**
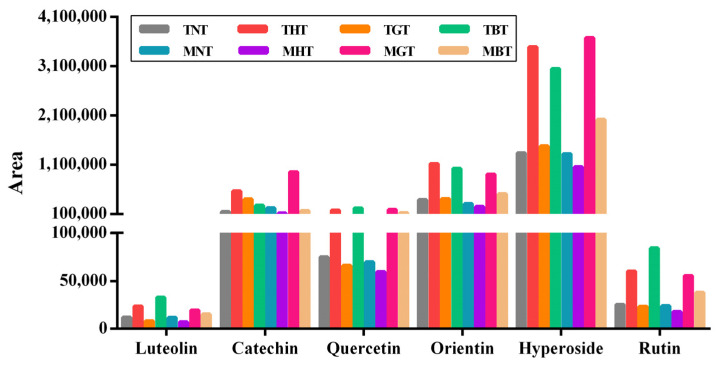
Comparison of the contents of luteolin, catechin, quercetin, orientin, hyperoside and rutin in lotus-leaf teas.

The content of polyphenols in lotus-leaf teas with different maturities in different processing method is shown in Figure 6. For the mature lotus-leaf tea, the levels of luteolin, quercetin, orientin, hyperoside and rutin were demonstrated as follows, MGT > MBT > MNT > MHT. However, the content of catechin was MGT > MNT > MBT > MHT. For the tender lotus-leaf tea, the THT group had a higher content of catechin, orientin, hyperoside and rutin than other groups, and the content of luteolin and quercetin were ranked only second to TBT group. TGT and TNT groups had a lower content of luteolin, quercetin, orientin, hyperoside and rutin than TBT and THT groups. The TGT group had a higher content of catechin than the TBT and TNT groups.

## 4. Conclusions

In the present study, volatile flavor components, TPC, TFC and antioxidant activity of varied lotus-leaf tea (white tea, green tea and black tea) were investigated. We found that the leaf maturity stage and processing methods influenced the flavor, phytochemical profiles and antioxidant activity of lotus-leaf tea. Lotus leaves at the mature stage showed more volatile components than those at the tender stage. Lotus leaves treated with white-tea process had more aroma components than other processing methods. However, green-tea-process samples exhibited the highest levels of TPC and TFC. The aqueous extract of various lotus-leaf teas had good antioxidant activities, and the lotus leaf at tender stage treated with green-tea process showed the strongest antioxidant activity. Our results suggested that leaves at the mature stage produced lotus-leaf tea with more aroma components. The green-tea process and black-tea process were first applied to produce lotus-leaf tea, and the green-tea process for lotus-leaf tea had a potential effect on TPC, TFC and antioxidant activity. The volatile profiling revealed the aroma of different lotus-leaf teas and highlighted the sensory properties of lotus-leaf tea as functional foods.

## Figures and Tables

**Table 1:** Antioxidant contents of various lotus-leaf teas.

	TNT	THT	TGT	TBT	MNT	MHT	MGT	MBT
TPC (mg gallic acid/g lotus tea)	41.59 ± 0.50 ^A^	48.62 ± 0.40 ^B^	47.45 ± 0.24 ^C^	18.65 ± 0.13 ^D^	35.42 ± 0.29 ^E^	32.77 ± 0.61 ^F^	49.97 ± 0.37 ^G^	27.51 ± 0.40 ^H^
TFC (mg rutin/g lotus tea)	57.25 ± 0.51 ^A^	70.09 ± 0.86 ^B^	65.78 ± 2.71 ^C^	26.02 ± 0.46 ^D^	52.67 ± 0.82 ^E^	49.87 ± 0.89 ^F^	73.43 ± 1.30 ^G^	43.02 ± 1.12 ^H^

Values not sharing a common letter (^A,B,C,D,E,F,G,H^) differ significantly among groups at *p* < 0.05.

**Table 2:** Identification of compounds in lotus-leaf teas with different maturities in different processing method.

No.	Formula	Molecular Weight (g/mol)	Retention Time (min)	Typical MS/MS Ions (*m*/*z*)	Identification
1	C_15_H_10_O_6_	286.04	4.33	151.00, 133.06, 107.01	Luteolin
2	C_15_H_14_O_6_	290.07	3.01	245.89	Catechin
3	C_15_H_10_O_7_	302.03	4.08	273.06, 257.04, 179.03, 164.03	Quercetin
4	C_21_H_20_O_11_	448.09	4.31	357.07, 327.08, 299.05	Orientin
5	C_21_H_20_O_12_	464.09	4.07	301.05, 300.05, 271.02, 255.82	Hyperoside
6	C_27_H_30_O_16_	609.14	3.95	301.05, 300.05	Rutin

## Data Availability

Not applicable.
